# Effectiveness of Australia’s Get Healthy Information and Coaching Service®: maintenance of self-reported anthropometric and behavioural changes after program completion

**DOI:** 10.1186/1471-2458-13-175

**Published:** 2013-02-26

**Authors:** Blythe J O’Hara, Philayrath Phongsavan, Elizabeth G Eakin, Elizabeth Develin, Joanne Smith, Mark Greenaway, Adrian E Bauman

**Affiliations:** 1 Prevention Research Collaboration, Sydney School of Public Health, Medical Foundation Building, University of Sydney, Sydney, NSW, 2006, Australia; 2 Cancer Prevention Research Centre, School of Population Health, Herston Road, Herston, University of Queensland, Queensland, 4006, Australia; 3 NSW Ministry of Health, 73 Miller Street, North Sydney, NSW, 2060, Australia

## Abstract

**Background:**

The Get Healthy Information and Coaching Service® (GHS) is a population-wide telephone-based program aimed at assisting adults to implement lifestyle improvements. It is a relatively uncommon example of the translation of efficacious trials to up-scaled real-world application. GHS participants who completed the 6-month coaching program made significant initial improvements to their weight, waist circumference, Body Mass Index (BMI), physical activity and nutrition behaviours. This study examines the maintenance of anthropometric and behaviour change improvements 6-months after program completion.

**Methods:**

GHS coaching participants (n=1088) were recruited between February 2009 and June 2011. Participants were eligible if they completed the 6-month coaching program and had available data at 12-month follow-up (n=277). Weight, waist circumference, BMI, fruit and vegetable consumption and physical activity were collected at baseline and 6-months by GHS coaches and 12-months (6-months post program) by independent evaluators. Matched pair t-tests, mixed linear regression and logistic regression analyses were performed to assess maintenance of program effects.

**Results:**

Improvements in weight (−2.9 kg, 95% CI: -3.6, -2.1), waist circumference (−5.4 cm, 95% CI: -6.7, -4.1), BMI (−1.1units, 95% CI: -1.5, -0.8), and fruit (+0.3 serves per day, 95% CI: 0.2, 0.3) and vegetable (+0.5 serves per day 95% CI: 0.3, 0.6) consumption were observed from baseline to 12-months. Apart from vegetable consumption, there were no significant differences between 6-month and 12-month changes from baseline, indicating these risk factor improvements were maintained from the end of the coaching program. There were also improvements in the proportion of participants undertaking recommended levels of physical activity from baseline to 12-months (increase of 5.2%), however the improvements made at end of the coaching program were not maintained at the 6-month follow up.

**Conclusions:**

This study provides preliminary evidence that the GHS has potential to contribute to substantial improvements in the chronic disease risk factor profile of program completers and facilitates sustained maintenance six months after completing the coaching program.

## Background

Obesity contributes to a significant number of chronic diseases and conditions, and with obesity prevalence continuing to rise [1], the implementation of population wide initiatives that impact on overweight and obesity, and accordingly the chronic disease risk factor profile of the community is a priority. The telephone-based Get Healthy Information and Coaching Service® (GHS) is one such initiative, introduced by the government in February 2009 in the state of New South Wales, Australia, aimed at assisting adults to make lifestyle improvements. The GHS provides information and a 6-month coaching program for participants free of charge.

The GHS represents the translation of efficacious trials to a real world population-level program. There is growing acceptance that the efficacy evidence for telephone-based lifestyle interventions is strong and that more trial evidence is no longer required. There is also increasing acknowledgement that the current evidence is limited on the practice of up-scaling and implementing interventions such as the GHS in the population health setting, and the short and longer term outcomes that can be achieved [2,3]. Previous research has demonstrated the effectiveness of the GHS coaching program on anthropometric and behavioural risk factor measures at program completion (ie: the short term) [4], confirming that results observed in the precursor trials [5-12] can be reproduced in population-based, translational context [13]. However, much less evidence is available as to whether programs such as GHS can promote the maintenance of behaviour change once the program has ceased [3]. Two telephone-based physical activity programs delivered and evaluated in applied settings have reported on maintenance effects [14,15]. Aside from these, the studies that report on the long-term maintenance of behaviour change have been limited to telephone based within controlled settings and often with a population of adults with particular chronic diseases [16] or within workplaces [7] as opposed to the general population in a real-world setting. This paucity of evidence is apparent within trials of telephone-based programs, but also in physical activity and nutrition intervention trials more generally, where a recent systematic review found that only 35% of included studies reported on maintenance of outcomes following the end of program [17]. This is compounded by telephone counselling itself frequently being used as a strategy for maintaining behaviour change after a more intensive intervention [16,18,19].

Within the context of the translation of trial evidence to a population-wide telephone-based coaching service, this study determines whether anthropometric and behavioural changes for a cohort of GHS participants are maintained 6-months following completion of the 6-month coaching program. It examines the magnitude of these changes, the proportion of participants who maintained improvements, and the socio-demographic factors associated with maintained improvements.

## Methods

### Study design and samples

Elements of the GHS, including evaluation methods and participant recruitment have previously been reported [20]. Briefly, this dissemination study employed a pre- and post-test evaluation design, and comprised two cohorts: the main GHS coaching cohort (with data collected by GHS coaches at baseline and 6-months) and the independent sub-sample coaching cohort, randomly drawn from the main GHS coaching cohort (with 12-months data collected by an independent evaluator).

**Figure 1:**
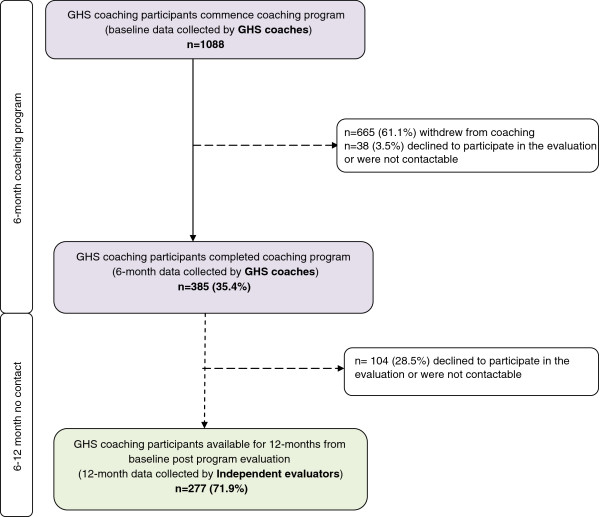
GHS Participant inclusion flow chart.

This study includes a sample of GHS participants who enrolled in the coaching program (n=1088) and who were randomly selected and invited to take part in the independent sub-sample cohort for the 12-month follow up study by the independent evaluator. A weekly rolling recruitment method was used to compile this sub-sample, where a random sample of 10% of GHS coaching participants were drawn and invited to take part in the study each week, except for those weeks where number of participants joining the coaching program were less than 20 and in this instance all of these participants were contacted to take part in the study. From this sub-sample those who had completed the 6-month coaching program, had data available at baseline, at 6-months (as collected by the GHS coaches) and at the 12-month follow-up (6-months after completion of the coaching program) were included in the analyses (Figure 1). Ethics approval for this study was granted by the University of Sydney Human Research Ethics Committee (Ref. No. 02-2009/11570).

### Measures

#### Socio-demographic variables

All measures were collected using computer-assisted telephone interviews (CATI) by GHS coaches during GHS program delivery and the independent evaluator. Data on gender, date of birth, residential postcode, education level [21], employment status, language spoken at home and Indigenous status were collected using questions from the NSW Population Health Survey [22]. Participants’ postcodes were used to determine Socio-Economic Indexes for Areas (SEIFA) [23], as a measure of area socio-economic status, and Accessibility-Remoteness Index of Australia Plus (ARIA) as a measure of geographical location remoteness [24].

#### Outcome measures

The primary anthropometric measures were self-reported weight (kg), height (cm), and waist circumference (cm), which were asked using a standard script, and where possible participants were instructed on how to collect these measurements and/or were provided with a tape measure for waist circumference. A subsample (n=38) recruited for a measurement validation sub-study [4,25] to compare self-report and objectively-measured weight, height and waist circumference indicated a moderate-strong correlation between self-report and objective anthropometric measurements (spearman rho > 0.9). Self-reported weight was 1.6 kg (95% CI: 0.8 kg to 2.4 kg) lower than objectively-measured weight and there was 84% and 87% agreement in Body Mass Index (BMI, kg/m2) and waist circumference classifications, respectively. Overall, these self-reported measurement errors are modest [26] and support the use of self-reported weight and height in this evaluation.

Height and weight were used to calculate BMI and then classified into: underweight (<18.49), acceptable weight (18.5—24.99), overweight (25.00—29.99) and obese (≥30.00) [1]. Waist circumferences risk categories were calculated for males (< 94 cm no risk; increased risk ≥94 cm to <102 cm; greatly increased risk ≥102 cm); and for females (< 80cm no risk; increased risk ≥80 cm to <88 cm; and greatly increased risk ≥88 cm) [27].

To minimise respondent burden, physical activity for baseline and 6-months (collected by GHS coaches), were assessed by three validated questions (3Q-PA), which asked about number of weekly walking sessions, moderate-intensity physical activity for 30 minutes or more; and vigorous-intensity physical activity for 20 minutes or more [28-32]. Categories for recommended physical activity were defined by those engaging in ≥5 sessions per week of walking, or ≥5 sessions per week of moderate activity, or combinations of walking and moderate-vigorous activity summing to 5 sessions per week [29]. For physical activity data at 12-months (collected by independent evaluator), the Active Australia Questionnaire (AAQ) was used which asked about the number of times participants walked, did moderate physical activity and/or vigorous activity; and the time spend on each of these activities [33]. Categories for recommended physical activity were defined as participants who had completed 150 minutes or more of physical activity, such that physical activity was defined as the combined time spent walking and engaging in moderate physical activity per week and vigorous activity (doubled) and combinations of 5 sessions per week [33]. A variable that reflects the health enhancing physical activity benefits was also computed to provide for comparability between the 3Q-PA and the AAQ, whereby the number of walking sessions and moderate physical activity sessions per week that were on average ≥30 minutes; and the number of vigorous physical activity sessions per week that were on average ≥20 minutes were summed together to create health enhancing physical activity sessions indicator.

For fruit and vegetable consumption at baseline, 6-months and 12-months, participants reported consumption of the number of daily serves of fruit and vegetables [34,35] to both GHS coaches and the independent evaluator. Participants were categorised into those meeting the recommended levels of consumption of ≥2 serves of fruit daily, and ≥5 serves of vegetables daily in accordance with Australian Dietary Guidelines [36].

### Data management and statistical analysis

Descriptive and chi square analyses were performed (IBM SPSS Inc. 2009) on key socio- demographic variables stratified by program time-period (baseline, 6-months and 12-months). Matched (within-individual) paired t-tests were performed to examine changes in weight, waist, and BMI from baseline to follow-ups, as these data followed normal distributions. Wilcoxon signed-rank tests were performed to examine changes in fruit and vegetable intake as these data were non-normally distributed.

For weight, BMI, waist circumference, fruit and vegetable variables, mixed linear regression models were used to examine changes between baseline and 12-months and between baseline and 6-months, adjusted for baseline levels [37], age, gender, education level, employment status, SEIFA and region. The models were limited to participants with data for at least one follow-up (i.e. either 6-months, 12-months or both). For the categorical variable of recommended physical activity, mixed generalized linear regression models [38] were used to examine changes between baseline, 6-months and 12-months, adjusted for age, gender, education level, employment status, SEIFA and region.

To allow for possible maintenance effects bias intention-to-treat analysis (ITT) was also performed, using the last known data observed to impute for missing follow-up data points.

Maintenance variables were created for all anthropometric variables. Participants were classified as having maintained or continued making improvements by first computing their change score from baseline to follow-ups [(Δ 12months - baseline) – (Δ 6 months - baseline)] and where the result was ≤0 kg change for weight and ≤0 cm for waist circumference, ≥0 daily serves of fruit and vegetables and ≥0 sessions of health enhancing physical activity. This method of reporting maintenance has been undertaken in the absence of a consensus on what constitutes weight-related behaviour change maintenance and allows for the analysis of socio-demographic characteristics of those defined as having maintained improvements. Logistic regression models were computed to examine the association between maintenance and continued improvements in weight and waist circumference and socio-demographic variables.

## Results

Between April 2009 and February 2011, a total of 1088 participants consented to take part in the long-term follow-up and provided baseline data; 385 (35.4%) completed the coaching program; and 277 of these (71.9%) were successfully followed up at 12-months (Figure 1).

### Participants’ socio-demographics, anthropometric and behavioural risk factor profile

Table 1 presents the socio-demographic, anthropometric and behavioural risk factor characteristics of participants included in the study at baseline, 6-months and 12-months. Based on a participants baseline characteristics, the majority of participants were female; aged 40 years +; had tertiary qualifications; employed full-time, spoke English; non-Aboriginal; had an income lower than $AUS80,000; were in the lowest three quintiles of advantage; lived in major cities; and did not require general practitioner consent to enrol in the coaching program. In terms of behavioural risk factor characteristics, coaching participants were classified as being overweight or obese according to their BMI, had a greatly increased risk of chronic disease due to their waist circumference measurement, were engaging in insufficient physical activity and did not consume the recommended number of fruit or vegetable servings. A comparison of the participants whose data were included at baseline, 6-months and 12-months showed that there those aged less than 50 years; were from major cities; and did not require medical clearance were less likely to have completed the coaching. There were no other significant differences on socio-demographic and behavioural risk factor baseline profile between participants over time. Further analysis comparing participants lost to follow up and those in this study revealed that participants with a higher body weight at baseline were more likely to be lost to follow up and there was a tendency for younger employed participants to also be lost to follow up (data not shown).

**Table 1:** **Socio**-**demographic**, **anthropometric and behavioural risk factor characteristics of GHS participants at baseline**, **six and twelve months** (**recruited between April 2009** – **February 2011**)

		**Baseline**		**Six months**			**Twelve month**		
		**n=1088**		**n=385**		**p-value†**	**n=277**		**p-value†**
		**n**	**%**	**n**	**%**		**n**	**%**	
**Gender**	Male	219	20.1	88	22.9	NS	58	20.9	NS
	Female	869	79.9	297	77.1		219	79.1	
**Age Groups**	18-49 years	647	59.5	186	48.0	**	134	48.4	NS
	50+ years	441	40.5	199	52.0		143	51.6	
**Education**~	Year 12 and below	367	33.9	129	33.6	NS	103	37.3	NS
	Other	717	66.1	255	66.4		173	62.7	
**Employment**	Employed (full, part time or casual)	720	66.4	257	66.8	NS	191	69	NS
	Other	365	33.6	128	33.2		86	31	
**Language**	English	1040	95.6	374	97.1	NS	270	97.5	NS
	Other	48	4.4	11	2.9		7	2.5	
**Indigenous**	Non Aboriginal	1062	97.6	379	98.4	NS	272	98.2	NS
	Aboriginal	26	2.4	6	1.6		5	1.8	
**Income**	<$AUS 80,000	556	58.1	206	59.9	NS	139	56.7	NS
	>$AUS 80,000	401	41.9	138	40.1		106	43.3	
**SEIFA**	1st, 2nd & 3rd quintiles (least advantaged)	595	57.4	203	54.1	NS	147	54.6	NS
	4th & 5th quintile (most disadvantaged)	441	42.6	172	45.9		122	45.4	
**Region**	Major Cities	595	57.5	184	49.1	*	140	52	NS
	Other	440	42.5	191	50.9		129	48	
**Coaching type**	No GP consent required	745	68.5	235	61.0	*	170	61.4	NS
	GP consent required	343	31.5	150	39.0		107	38.6	
**Body Mass Index** (**BMI**) **classifications**	Under & acceptable weight (BMI 10.0-24.99)	162	16.2	67	18.1	NS	54	20.3	NS
	Overweight & Obese (BMI 25.0+)	835	83.8	303	81.9		212	79.7	
**Waist circumference risk** ¥	No risk	77	10.2	33	10.6	NS	26	11.5	NS
	Increased & greatly increased risk	679	89.8	282	89.4		201	88.5	
Recommended physical activity***	Insufficient	663	65.7	231	61.8	NS	163	60.8	NS
	Sufficient	346	34.3	142	38.2		105	39.2	
**Fruit and vegetable consumption**	Consumes <2 serves of fruit daily	479	47.4	172	46.2	NS	124	46.3	NS
	Consumes ≥2 serves of fruit daily	531	52.6	200	53.8		144	53.7	
	Consumes <5 serves of vegetables daily	859	85	313	83.7	NS	222	82.8	NS
	Consumes ≥5 serves of vegetables daily	151	15	61	16.3		46	17.2	

***Notes***:

† Chi-square tests of significance between baseline and six months, and six months and twelve months.

~ Year 10 or below is the equivalent of lower secondary education; Year 12 or below is the equivalent of upper secondary education; Diploma & Certificate is the equivalent of post secondary non tertiary education; and degree or higher is the equivalent of tertiary qualifications [21].

¥ Waist circumference risk: no risk is ≤80 cm for women and ≤94 cm for men; increased risk is between 81 and 88 cm for women, and between 95 and 102 cm for men; greatly increased risk >88 cm for women and >102 cm for men [27].

*** Recommended Physical Activity: ≥5 sessions per week walking or ≥5 session per week of moderate activity, or 3–4 sessions per week walking and 1–2 sessions per week of moderate activity, or ≥1-2 sessions per week walking and 3–4 sessions per week of moderate activity [29].

* p-value <0.05; ** p-value <0.001.

### Maintenance of anthropometric and behavioural risk factors changes

Table 2 shows changes from baseline to 6-months (end of coaching), changes from baseline to 12-months follow-up, and changes between 6-and 12-months (no contact period). Adjusted mean change from baseline to 12-months showed improvements in weight of −2.9 kg (95% CI: -3.6, -2.1); waist circumference of −5.4 cm (95% CI: -6.7, -4.1); BMI of −1.1 kg/m2 (95% CI: -1.5, -0.8); fruit of +0.3 daily serves (95% CI: 0.2, 0.3); and vegetables of +0.5 daily serves (95% CI: 0.3, 0.6). Adjusted mean differences between 6-months and 12-months indicate that improvements made at 6-months following the end of coaching were maintained at 12-months, with no significant variations for weight, waist circumference, BMI, and daily fruit servings. In relation to daily vegetable servings the adjusted mean difference showed a significant decrease of 0.5 serves between 6-months and 12-months (p<0.001). In relation to health enhancing physical activity sessions, the adjusted mean difference showed an increase of 1.8 sessions between baseline and 6-months (p<0.001), and an increase of 1.2 sessions between 6- and 12-months (p<0.05).

**Table 2:** **Anthropometric**, **fruit and vegetable consumption changes from baseline to six months and twelve** (**Coaching Period**), **and from six months to twelve months** (**No Contact Period**)

	**COACHING PERIOD**				**NO CONTACT PERIOD**							
	**6**-**month**				**12**-**month**				**6**-**12 months†**			
	**(end of coaching) †**				**(maintenance follow**-**up) †**							
	**Baseline**	**6-month**	**Mean change**	**Adjusted € change (95% CI)**	**Baseline**	**12-month**	**Mean change**	**Adjusted € change (95% CI)**	**6-month**	**12-month**	**Mean change**	**Adjusted € change (95% CI)**
**Weight(kg)¥**	n=357				n=257				n=251			
Mean	83.8	80.1	−3.6**	−3.6	82.0	79.2	−2.8**	−2.9	78.3	79.0	0.7*	0.7
(SD)	(18.4)	(17.3)	(4.9)	(−4.0, -3.1)	(16.6)	(16.5)	(6.3)	(−3.6, -2.1)	(15.6)	(16.5)	(4.2)	(−0.2, 1.6)
**Waist Circumference(cm)¥**	n=275				n=185				n=184			
Mean	99.1	94.7	−4.4**	−4.3	98.2	92.9	−5.3**	−5.4	93.6	92.3	−1.3*	−1.1
(SD)	(13.1)	(12.6)	(5.4)	(−4.9, -3.7)	(12.9)	(13.9)	(9.0)	(−6.7, -4.1)	(12.6)	(13.5)	(8.4)	(−2.5, 0.3)
**Body Mass Index(kg/m**^**2**^**)¥**	n=357				n=257				n=250			
Mean	30.1	28.8	−1.3**	−1.3	29.7	28.6	−1.1**	−1.1	28.4	28.6	0.2	0.2
(SD)	(6.1)	(5.7)	(1.8)	(−1.5, -1.1)	(5.7)	(5.4)	(3.0)	(−1.5, -0.8)	(5.3)	(5.4)	(2.1)	(−0.2, 0.6)
Mean	1.7	1.9	0.2**	0.3	1.7	1.9	0.2**	0.3	1.9	1.9	0.0	0.0
(SD)	(1.2)	(0.8)	(1.1)	(0.2, 0.3)	(1.1)	(0.9)	(1.1)	(0.2, 0.3)	(0.8)	(0.9)	(0.9)	(−0.1, 1.1)
**Vegetable serves per day#**	n=371				n=264				n=264			
Mean	2.9	3.9	1.0**	0.9	3.0	3.4	0.4**	0.5	4.0	3.4	−0.6**	−0.5**
(SD)	(1.6)	(1.3)	(1.3)	(0.8, 1.1)	(1.6)	(1.5)	(1.8)	(0.3, 0.6)	(1.3)	(1.5)	(1.6)	(−0.7,-0.3)
**Health enhancing PA(sessions)#β**	n=371				n=245				n=244			
Mean	4.1	5.8	1.8**	1.8	4.3	5.5	1.2*	1.2	6.2	5.5	−0.7*	−0.6
(SD)	(3.9)	(3.7)	(4.3)	(1.4, 2.2)	(3.9)	(5.7)	(6.3)	(0.4,2.0)	(3.5)	(5.7)	(5.7)	(−1.5,0.3)

***Notes***:

** significant at p-value<0.001.

* significant at p-value <0.05.

Ŧ Baseline and six month data is sourced from the GHS coaching sample and the twelve month data is sourced from independent evaluation coaching cohort data.

† Paired analysis; ¥ Matched pairs *t*-test; ^#^ non parametric (wilcoxon test).

**β** Health enhancing Physical Activity (PA) sessions – based on the number of sessions per week for walking and moderate physical activity that were ≥30minutes and for vigorous physical activity that were ≥ 20minutes.

€ Adjusted for baseline values and age, gender, education level, employment status, Socio Economic Index For Areas, and Region; presents marginal means (or mean differences) with 95% Confidence Interval (CI) from linear mixed models that control for repeated measures and adjust for potential confounders, with the outcome time points of 6-months and 12-months. Models are limited to participants with data for at least one follow-up (i.e. either 6-months, 12-months or both).

Further, in relation to physical activity, after adjusting for socio-demographic variables, participants were significantly more likely to meet guidelines of recommended physical activity at 6-months (AOR: 3.23, p<0.001) compared to baseline and there was an increased likelihood of participants meeting guidelines of recommended physical activity at 12-months compared to baseline (AOR: 1.68, p=0.055) although this was not significant (data not shown).

The direction of improvements as detailed in Table 2 remained the same when we re-computed the analyses using intention-to-treat approach, although the magnitude of change was considerably smaller (Table 3).

**Table 3:** **Intention**-**to**-**treat analysis**: **Anthropometric**, **fruit and vegetable consumption changes from baseline to six months** (**coaching period**) **and twelve**, **and from six months to twelve months** (**no contact period**)

	**COACHING PERIOD**				**NO CONTACT PERIOD**							
	**6**-**month**				**12**-**month**				**6**-**12 months†**			
	**(end of coaching) †**				**(maintenance follow**-**up) †**							
	**Baseline**	**6-month**	**Mean change**	**Adjusted € change (95% CI)**	**Baseline**	**12-month**	**Mean change**	**Adjusted € change (95% CI)**	**6-month**	**12-month**	**Mean change**	**Adjusted € change (95% CI)**
**Weight** (**kg**)¥	n=1000				n=1000				n=1006			
Mean	86.5	84.7	−1.8**	−1.8	86.5	84.9	−1.6**	−1.6	84.7	84.9	0.2*	0.2
(SD)	(20.0)	(19.6)	(3.9)	(−2.1,-1.6)	(20.0)	(19.8)	(4.3)	(−1.9,-1.4)	(19.7)	(19.8)	(2.2)	(−0.2,0.5)
**Waist Circumference** (**cm**)¥	n=756				n=756				n=806			
Mean	101	98.8	−2.2**	−2.2	101	98.4	−2.6	−2.6**	98.6	98.3	−0.3*	−0.3
(SD)	(15.8)	(15.9)	(4.3)	(−2.5,-1.9)	(15.8)	(16.3)	(5.8)	(−3.0,-2.2)	(15.8)	(16.1)	(4.2)	(−0.8,0.2)
**Body Mass Index** (**kg**/**m**^**2**^)¥	n=998				n=998				n=1001			
Mean	31	30.5	−0.5**	−0.5	31	30.5	−0.5**	−0.5	30.6	30.5	−0.1	−0.1
(SD)	(6.4)	(6.4)	(1.2)	(−0.5,-0.4)	(6.4)	(6.4)	(1.8)	(−0.7,-0.4)	(6.4)	(6.4)	(1.4)	(−0.2,0.1)
**Fruit serves per day**#	n=1010				n=1011				n=1011			
Mean	1.7	1.8	0.1**	0.1	1.7	1.8	0.1**	0.1	1.8	1.8	0.0	0.0
(SD)	(1.3)	(1.1)	(0.8)	(0.1, 0.2)	(1.3)	(1.1)	(0.8)	(0.1,0.2)	(1.1)	(1.1)	(0.5)	(−0.1,0.1)
**Vegetable serves per day**#	n=1010				n=1010				n=1011			
Mean	2.8	3.3	0.5**	0.5	2.8	3.2	0.4**	0.4	3.3	3.2	−0.2**	−0.1
(SD)	(1.6)	(1.5)	(1.2)	(0.4,0.6)	(1.6)	(1.5)	(1.3)	(0.3,0.4)	(1.5)	(1.5)	(0.9)	(−0.2,0.1)
**Health enhancing PA** (**sessions**)#**β**	n=1010				n=1010				n=1011			
Mean	3.9	4.6	0.7**	0.7	3.9	4.4	0.5**	0.5	4.6	4.4	−0.2*	−0.2
(SD)	(3.9)	(3.9)	(2.8)	(0.5,0.8)	(3.9)	(4.5)	(3.5)	(0.3,0.7)	(3.9)	(4.5)	(2.8)	(−0.4,0.1)

***Notes***:

** significant at p-value<0.001; * significant at p-value <0.05.

Ŧ Baseline and six month data is sourced from the GHS coaching sample and the twelve month data is sourced from independent evaluation coaching cohort data.

£ Includes data imputed from last observed record (baseline, 3-months or 6-months) where data was missing.

† Paired analysis; ¥ Matched pairs *t*-test; ^#^ non parametric (wilcoxon test).

β Health enhancing Physical Activity (PA) sessions – based on the number of sessions per week for walking and moderate physical activity that were ≥30minutes and for vigorous physical activity that were ≥ 20minutes.

€ Adjusted for baseline values and age, gender, education level, employment status, Socio Economic Index For Areas, and Region; presents marginal means (or mean differences) with 95% Confidence Interval (CI) from linear mixed models that control for repeated measures and adjust for potential confounders, with the outcome time points of 6-months and 12-months.

In relation to the proportion of participants at 12-months follow-up [6-months after end of coaching], who maintained or continued to make improvements 49.0% of participants did so in regard to their weight; 63.0% in regard to their waist circumference; 69.2% did so in relation to their fruit consumption and 45.6% did so in relation to vegetable consumption (data not shown).

Table 4 shows predictors of behavioural maintenance. Results showed that participants from locations other than major cities were less likely to maintain or continue to make improvements with respect to weight (AOR: 0.5, 95% CI: 0.3, 1.0). Gender, age, education level, employment status and socio-economic status were not significantly associated with maintaining or continuing to reduce weight and waist circumference.

**Table 4:** **Adjusted Odds Ratio** (**AOR**) **and 95% confidence intervals** (**CI**) **for likelihood of** "**maintaining or continuing weight and waist circumference reductions**" **six months after end of coaching**

		**Weight maintenance**					**Waist circumference maintenance**				
		**N**	**n**	**%**	**AOR(95% CI)**	**p**-**value**	**N**	**n**	**%**	**AOR(95% CI)**	**p**-**value**
**Gender**	Male (ref)	57	24	42.1			40	24	60.0		
	Female	192	98	51.0	1.4 (0.8, 2.7)	0.238	122	78	63.9	1.1 (0.5, 2.3)	0.798
**Age Groups**	18-49 years (ref)	117	57	48.7			72	40	55.6		
	50+ years	132	65	49.2	1.0 (0.6,1.7)	0.911	90	62	68.9	1.5 (0.7,3.1)	0.247
**Education**	High school education (ref)	91	49	53.8			58	43	74.1		
	Certificate/Degree/Higher	157	72	45.9	0.7 (0.4,1.2)	0.161	103	58	56.3	0.5 (0.3,1.2)	0.135
**Employment**	Full time/part time/Casual (ref)	171	82	48.0			108	63	58.3		
	Other	78	40	51.3	1.0 (0.5,1.8)	0.989	54	39	72.2	1.2 (0.5, 2.8)	0.637
**SEIFA**	1st, 2nd, quintile - most advantaged (ref)	138	68	49.3			94	57	60.6		
	3rd 4th & 5th-quintile-most disadvantage	111	54	48.6	1.1 (0.6,2.0)	0.705	68	45	66.2	1.0 (0.5,2.1)	0.999
**Region**	Major Cities (ref)	130	70	53.8			83	49	59.0		
	Other	119	52	43.7	0.5 (0.3,1.0)	0.043	79	53	67.1	1.3 (0.6, 2.7)	0.504

***Notes***:

Ref: reference group.

Maintenance or continuation of weight reductions calculated by (Δ 12months - baseline) – (Δ 6 months - baseline) defined as change in weight ≤0kg.

Maintenance or continuation of waist circumference reductions calculated by (Δ 12months – baseline) – (Δ 6 months - baseline) defined as change in waist circumference ≤0cm.

### Changes in classifications for BMI, waist circumference, daily fruit and vegetable consumption and physical activity

**Figure 2:**
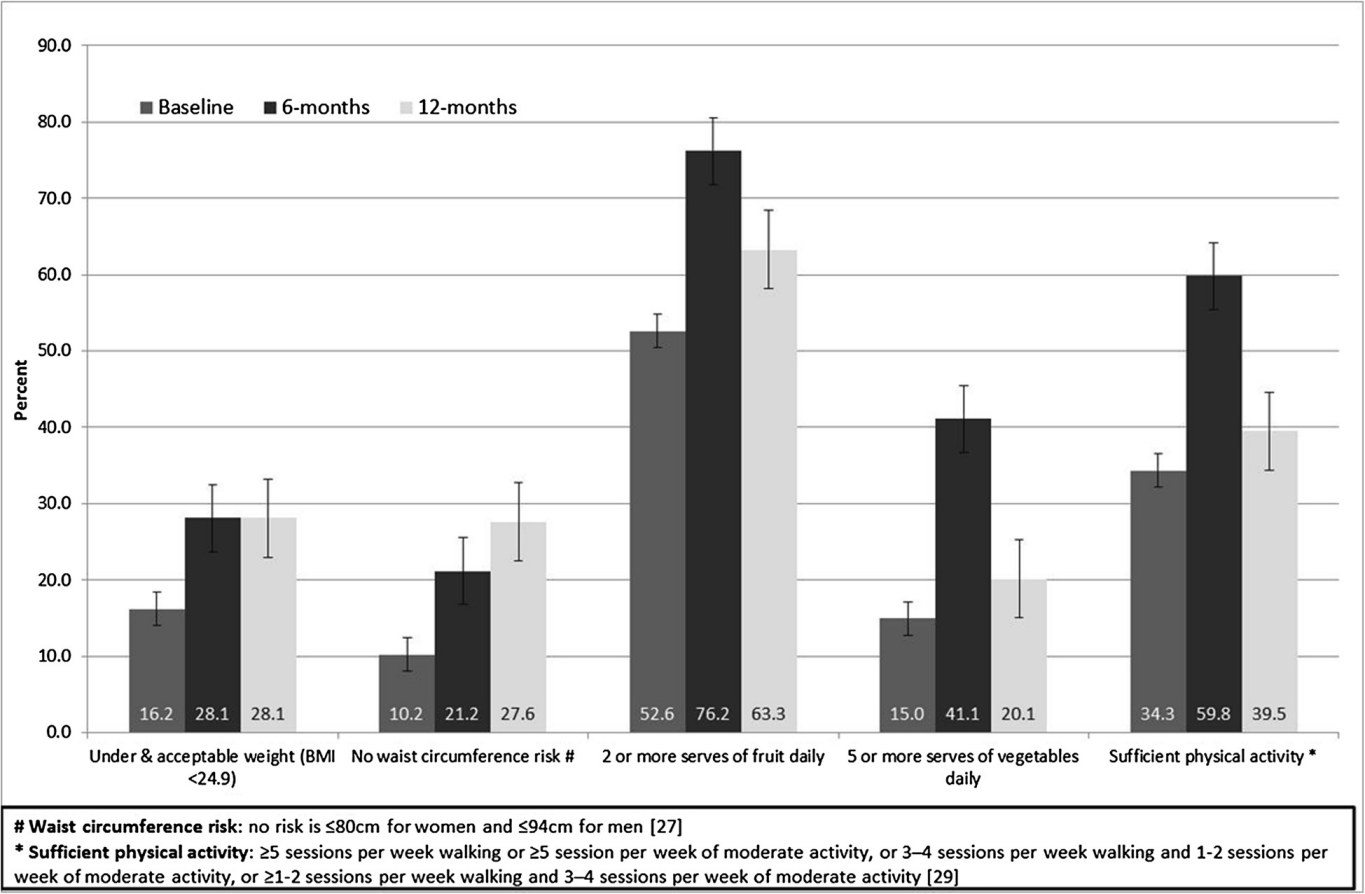
**Proportions in classifications of Body Mass Index(BMI);waist circumference risk;fruit and vegetable consumption and physical activity at baseline,six months and twelve months.**

Figure 2 presents baseline, 6- and 12-months data according to categorical classifications of anthropometric and behavioural risk factors. For BMI, higher proportions of participants were classified as being an acceptable weight at 6-months (11.9% increase) and this proportion was maintained at 12-months; the proportions categorised as being of no increased waist circumference risk increased by 11.0% at 6-months and was also maintained at 12-months. The proportion of participants meeting the recommended levels of daily fruit and vegetables consumption was not maintained from 6-months to 12-months but was still an improvement on baseline levels. For physical activity, there were greater proportions of participants engaging in recommended levels of physical activity at 6-months compared to baseline (25.5% increase), however, this was not maintained at 12-months.

## Discussion

This study reported on the maintenance of self-reported anthropometric and behavioural risk factors changes, 6-months post program for a small (n=277) cohort of individuals participating in and completing a population-wide translational behaviour change program. Effects were observed as being maintained for both anthropometric measures and risk factor behaviours (aside from vegetable consumption). As one of the few population-wide telephone coaching programs that reports on the maintenance of behaviour change after program completion, these results fill an important gap in the evidence base [3].

The results of this study demonstrate that GHS coaching participants experienced improvements in their weight, waist circumference and BMI from baseline to 12-months. Additionally, improvements were maintained during the no contact period from the end of coaching sessions for a further six months. That is, there was no significant increase in weight, waist circumference, BMI or decline in fruit consumption during the no contact period. Even after adjusting for baseline levels and socio-demographic variables, the program had significant maintenance effects for all anthropometric variables and for fruit (but not vegetable) consumption. While there were improvements from baseline to 12-months, at 12-months vegetable consumption had declined by 0.5 daily serves from the improvements achieved at the end of the 6-month coaching program. This is consistent with the results reported in another intervention maintenance study of a telephone-based program in the primary care setting [37].

As previously reported [4], GHS participants who completed the 6-months coaching program reported statistically significant improvements in weight (−3.9 kg); waist circumference (−5.0 cm); and BMI (−1.4 BMI units) from baseline to the completion of coaching [4]. The present study demonstrated the potential for these gains to have been maintained for a further 6-months post-program. Whilst further verification of these results with a larger sample size is warranted, early indications are that this could have important population-health implications for decreasing chronic disease risk factors, weight loss and waist circumference decreases, even at modest levels are beneficial for cancer, diabetes and cardiovascular disease risk reduction [1,39-44]. Conversely, increased body weight is strongly associated with increased risk of developing a number of chronic diseases and conditions. Consistent with this body of evidence, maintenance of weight and waist circumference loss (even at modest levels) can decrease chronic disease risk.

In relation to the maintenance of physical activity improvements, this study showed that participants increased the number of health enhancing physical activity sessions from baseline to 6-months and to 12-months, and that an additional 25.5% of participants were meeting recommended activity guidelines at the completion of the coaching program. These improvements were not maintained at 12-months from the levels obtained at 6-months, although there were still improvements from baseline with an increase of 5.2% participants meeting recommended physical activity guidelines. These results are consistent with findings in other small-scale and less generalisable studies that included a telephone coaching component [10,14]. Adjusted models confirmed this finding.

This study shows a high proportion of participants maintained or continued to make improvements to the lifestyle changes they had made at 6-months. Approximately half of the coaching participants included in this study maintained or continued to make weight improvements; nearly two-thirds maintained or continued to make waist circumference improvements; and maintained or continued to make fruit consumption improvements and approximately half maintained or continued to make vegetable consumption improvements. This is a positive outcome for the GHS suggesting the potential population impact that GHS might have, with nearly two-thirds of participants maintaining a lifestyle improvement, at least in the medium term.

There are sub-populations targets within the adult population who have a higher prevalence of lifestyle risk factors and higher incidence of chronic diseases and conditions [45,46] and accordingly would benefit from the services that GHS has to offer. The findings of this study suggest that participant socio-demographic profile did not influence the likelihood of maintaining or continuing weight or waist circumference improvements at the end of the coaching program, aside from those in major cities who were more likely to maintain or continue weight improvements compared to those who lived outside of major cities. This suggests that the GHS is equitable in terms of reaching at-risk populations from disadvantaged backgrounds and regional areas, [47] that the improvements maintained by GHS participants six months following program completion are unlikely to be influenced by socio-demographic characteristics [4].

The results reported in this study have some implications for on-going GHS implementation. Previous research has found that offering a telephone-based program that lasts more than 24 weeks and includes more than seven program contacts leads to better maintenance of behaviours [17]. The GHS coaching program provides an intervention of 26 weeks and includes 10 telephone contacts, the favourable maintenance results for those completing the 6-month coaching program further support previous research and also support the notion that the structure of the current coaching program itself is satisfactory. However, perhaps the greatest implication for the GHS and similar programs is what initiatives can be implemented to improve the retention and completion rate of GHS coaching participants; and thereby ensuring that a greater proportion of participants complete the coaching program and have the potential to benefit from achieving and maintaining changes in lifestyle behaviours. It is conceivable that with greater participation, retention and completion in the GHS coaching program a more substantial impact on chronic disease risk factors may be realised.

This study has some limitations due in part to its real world setting, particularly in relation to the attrition rate due to those not completing coaching, those lost to follow-up (uncontactable) or no longer wishing to be involved in the evaluation. However, the participants included in this maintenance study, firstly are similar in demographic profile to GHS participants overall [47], and secondly are similar in demographic and risk factor profile to those who were present in this study at both baseline and 6-months. The lost to follow-up in this study is not unexpected given the program is delivered without cost to participants, and noting relatively similar attrition rates experienced by other population-wide programs [48,49]. Whilst this significant loss to follow up is not ideal and would not be considered acceptable in efficacy trials, as a population- wide intervention with a greater degree of external validity the experience of the GHS highlights the complexities of undertaking such an evaluation, and the constant tension between scientific rigour and implementation research in the real world.

This study also presents results based on ITT analyses. It should be noted that while the ITT analyses may yield less biased point estimates, they are also likely to yield downward biased standard errors of program effects due to inflated correlations between subsequent assessments resulting from a large number of imputed data.

The reliance on self-report data [50] poses concerns in terms of socially-biased responses [51] and accuracy in reporting of anthropometric measures and behaviours [26]. Whilst findings from a measurement validation study conducted as part of the overall GHS evaluation [20,25] provide support for the acceptable reliability of self-reported height, weight and waist circumference categories, and the acceptable but modest validity of physical activity and nutrition variables is consistent with previous studies [52,53], the results should also be interpreted with some degree of caution.

Categorical variables for physical activity were also presented to further verify the results pertaining to health enhancing physical activity sessions as different instruments were used to collect data at baseline, 6-months and those used to collect data at 12-months. This meant that the analysis provided in relation to maintenance of physical activity is limited and the results presented in this study should be viewed with caution, future studies would warrant investigating this further.

Finally, evaluation of the GHS does not include a comparison group. While we recognise the value of randomised controlled trials as the gold-standard for assessing intervention effects, we contend that randomisation or a quasi-experimental with comparison groups are neither feasible nor appropriate in such an up-scaled setting (nor is it considered necessary given the irrefutable efficacy trials evidence in this field [3]). The GHS is a translational program being implemented across the whole population of adults, and accordingly we employed a feasible evaluation design [13,54] that strikes a balance between scientific rigours but yet respectful of the real-life application of complex programs such as the GHS. Considering alternative intervention evaluation designs for translational programs is also increasingly being recognised as valuable for providing policy- and practice-relevant evidence [55,56], as has also been illustrated through the experience of the GHS evaluation.

## Conclusions

As an effective population-wide program, the GHS provides preliminary evidence that it has potentials to contribute to substantial improvements in program completers’ chronic disease risk factor profile and facilitates sustained maintenance 6-months after the completion of the coaching program. An extended 2-year follow-up from baseline is recommended to determine whether long-term effects are still maintained.

## Competing interests

The authors declare that they have no competing interests.

## Authors' contributions

BOH was responsible for the design, drafting, analysis of data, and drafting and editing of the manuscript. PP was responsible for reviewing and editing the manuscript and overseeing the data analysis. EE was responsible for the reviewing and editing the manuscript and advising on statistical analysis. ED was responsible for the reviewing and editing the manuscript. JS was responsible for the reviewing and editing the manuscript. MG was responsible for confirming the results and reviewing and editing the manuscript. AB was responsible for the reviewing and editing the manuscript and advising on statistical analysis. All authors read and approved the final manuscript.

## Pre-publication history

The pre-publication history for this paper can be accessed here:

http://www.biomedcentral.com/1471-2458/13/175/prepub
